# Fabrication of Localized Surface Plasmon Resonance Fiber Probes Using Ionic Self-Assembled Gold Nanoparticles

**DOI:** 10.3390/s100706477

**Published:** 2010-07-01

**Authors:** Miao Wan, Pengfei Luo, Jiayi Jin, Jiong Xing, Zhiyong Wang, Stephen T. C. Wong

**Affiliations:** 1 Bioengineering and Bioinformatics Program, The Methodist Hospital Research Institute, Weill Cornell Medical College, Houston, Texas 77030, USA; E-Mails: wmwhdz07@gmail.com (M.W.); PLuo@tmhs.org (P.L.); JXing@tmhs.org (J.X.); 2 College of Math and Physics, China University of Geoscience, Wuhan 430074, China; 3 Department of Electrical & Computer Engineering, Texas A&M University, College Station, Texas 77844, USA; E-Mail: jjy2005@tamu.edu (J.J.)

**Keywords:** localized surface plasmon resonance, ionic self-assembled multilayers, fiber optics, biosensors

## Abstract

An nm-thickness composite gold thin film consisting of gold nanoparticles and polyelectrolytes is fabricated through ionic self-assembled multilayers (ISAM) technique and is deposited on end-faces of optical fibers to construct localized surface plasmon resonance (LSPR) fiber probes. We demonstrate that the LSPR spectrum induced by ISAM gold films can be fine-tuned through the ISAM procedure. We investigate variations of reflection spectra of the probe with respect to the layer-by-layer adsorption of ISAMs onto end-faces of fibers, and study the spectral variation mechanism. Finally, we demonstrated using this fiber probe to detect the biotin-streptavidin bioconjugate pair. ISAM adsorbed on optical fibers potentially provides a simple, fast, robust, and low-cost, platform for LSPR biosensing applications.

## 1. Introduction

Localized surface plasmon resonance (LSPR) is the resonance phenomenon of surface plasmons in the metal [1,2]. It occurs when light interacts with particles much smaller than the incident wavelength. Because LSPR is sensitive to changes in the local dielectric environment, it has been extensively studied for use in optical spectroscopy and biosensing applications [3]. In addition, because fiber optic biosensors have been attractive due to their advantages such as light, compact, remote sensing capability, and multiplexing capability, the combination of fiber optics and LSPR technique potentially can be used to construct high-sensitive, small biosensors for biosensing applications [4–7]. Usually people use either the covalent-bond self-assembly monolayer (SAM) technique or the lithography technique to immobilize or fabricate the gold nanoparticles (AuNPs) on the substrate [4,8]. However, SAM technique is time consuming and it is unable to make multilayer films, whereas the lithography technique is not only time consuming but also expensive.

The ionic self-assembled multilayers (ISAM) technique [9–16] provides a highly controllable means to build precise, nm-thick thin films on any charged surface. ISAM technique would be largely independent on the nature, size, and topology of the substrate. Various materials can be used as substrates such as glass, silicon, metal, cell, *etc.* Furthermore, a diverse array of materials, e.g., organic/inorganic/metal nanoparticles, proteins, and virus particles, can be incorporated into ISAM films. Hence, ISAM based gold films adsorbed on optical fibers can potentially provide a fast, cost effective, robust platform for building efficient LSPR sensors.

In this paper, we demonstrate that the LSPR spectrum induced by the ISAM based gold thin film can be fine-tuned through the ISAM procedure. An nm-thickness composite gold film consisting of AuNPs and polyelectrolytes is fabricated through ISAM technique and was deposited on end-faces of multimode optical fibers to construct LSPR fiber probes. We investigate variations of reflection spectra of the probe with respect to the layer-by-layer adsorption of ISAMs onto end-faces of optical fibers, and study the LSPR spectrum variation mechanism. Finally, we demonstrated using this fiber probe to detect the biotin-streptavidin bioconjugate pair. ISAM adsorbed on optical fibers potentially provides a simple, fast, robust, and low-cost, platform for LSPR biosensing applications.

## 2. Results and Discussions

### 2.1. Materials and Methods

**Figure 1.**
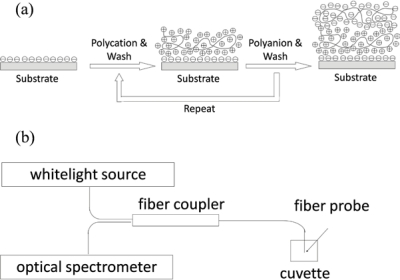
**(a)** ISAM film deposition process and illustration of nanofilm architecture of the first two layers (one bilayer); **(b)** Schematic of optical setup for experiments.

Colloidal AuNPs for synthesizing ISAM gold films were prepared using traditional Turkevich method through reducing gold (III) ion of hydrogen tetrachloroaurate (HAuCl_4_) to gold solid with sodium citrate as a reducing agent [17]. Powder of HAuCl_4_ and sodium citrate are purchased from Sigma-Aldrich Corp. 16nm-diameter AuNPs were used to fabricate ISAM films in our experiments, because smaller the size of AuNPs, more robust the assembled ISAM films. The ISAM deposition process, also known as layer-by-layer (LbL) deposition, is schematically shown in Figure 1a [9–16]. Through alternately immersing a charged substrate into anionic and cationic polyelectrolyte aqueous solutions, a nanoscale multilayer thin film is built due to electrostatic forces. The combination of one monolayer of polycation and polyanion is denoted a bilayer. In our experiments, an optical fiber was used as a substrate, citrate-anion coated colloid AuNPs were used as the polyanion while poly(allylamine hydrochloride) (PAH) at pH = 4.3 was used as the polycation. Bi-directional 1 × 2 50/125 multimode fiber couplers (Fiber Instrument Sales, Inc.) were used for reflection spectra measurements. The fiber of the coupler is Corning InfiniCor 600 graded-index multimode fiber. Its core diameter and cladding diameter are 50 μm and 125 μm respectively, numerical aperture is 0.2, and group index of refraction at 850 nm is 1.481. The optical source is HL-2000-HP Tungsten Halogen Source (Ocean Optics, Inc.). Reflection spectra were measured using an HR4000 Spectrometer (Ocean Optics, Inc.). The schematic of optical setup is shown in Figure 1b.

### 2.2. PAH/AuNPs ISAM Films on Fiber End-Face

**Figure 2.**
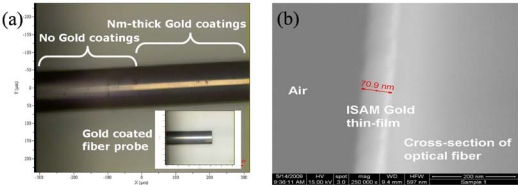
**(a)** Color picture of ISAM gold film coated fiber probe imaged by brightfield microscopy, inset is a cleaved fiber probe with gold coatings; **(b)** SEM picture of cross-section of a cleaved optical fiber coated by 10-bilayer ISAM gold films.

First, we demonstrated the feasibility of constructing nm-thick gold film coated fiber probes by depositing AuNPs onto fiber end-faces through ISAM process. We deposited 10-bilayer PAH/AuNPs films onto the fiber end-face to construct the fiber probe using 16 nm-diameter AuNPs. We alternately immersed the fiber probe into PAH and AuNPs solutions. Figure 2a shows the color picture of the ISAM gold film coated fiber probe imaged by brightfield microscopy. We can clearly see the gold metallic color in the portion of fiber with ISAM gold film coatings, and the inset exhibits a cleaved fiber probe with ISAM gold coatings. Figure 2b shows the SEM picture of cross-section of the ISAM gold film coated fiber after we cleaved the optical fiber.

**Figure 3.**
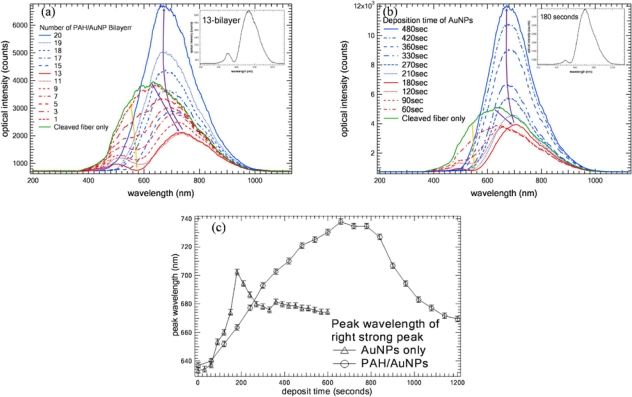
**(a)** Reflection spectrum of fiber probe as a function of number of coated PAH/AuNPs (16 nm) ISAM bilayers measured in air, AuNPs and PAH are alternately deposited every 30 seconds. Yellow arrow and purple arrow indicate the attenuation valley variation trend and the trend of the longer-wavelength strong peak shift as the number of bilayers increases, respectively; **(b)** After coated with one monolayer of PAH, fiber probe is immersed in AuNPs colloid solution. Reflection spectrum of fiber probe is measured every 30 seconds in air. Yellow arrow and purple arrow indicate the attenuation valley variation trend and the trend of the longer-wavelength strong peak shift as the number of bilayers increases, respectively; **(c)** Wavelength shifts of longer-wavelength strong peak in (a) and (b) as function of deposition time in PAH/AuNPs and AuNPs, respectively.

To construct a LSPR fiber probe for sensing, we studied the optical properties of the ISAM gold film coated fiber probe. We measured the reflection spectra of the probe after the deposition of each PAH/AuNPs (16 nm) bilayer. We alternately immersed the fiber probe into PAH and AuNPs (16 nm) every 30 seconds, which allows us to build a thinner ISAM film to more precisely reveal the evolution progress of reflection spectra. Figure 3a shows the reflection spectra as a function of the number of bilayers measured in air. We noted that after we cleaved the fiber, there was a weak broadband reflection spectrum which is determined by correlation between the whitelight source and the fiber transmission spectrum. As we coated the fiber layer by layer, there gradually appeared an attenuation valley around 570 nm due to the extinction of LSPR of AuNPs as shown in the Figure 3a. The attenuation valley splits the reflection spectrum into two peaks: one weak peak around 500 nm and one strong peak at longer wavelength. As bilayers increases, the longer-wavelength strong peak shifts to longer wavelength while its intensity decreases for the first 12 bilayers illustrated in Figure 3a and evaluated in Figure 3c. In Figure 3a, yellow arrow and purple arrow indicate the attenuation valley variation trend and the trend of the longer-wavelength strong peak shift as the number of bilayers increases, respectively. After the 12th bilayer, the strong peak shifts to shorter wavelength while its intensity gradually increases as bilayers increases illustrated in Figure 3a and evaluated in Figure 3c. The attenuation valley around 570 nm gradually smoothes and disappears.

To study the effect of PAH in PAH/AuNPs film, we first deposited one monolayer of PAH on the fiber end-face. Then, we immersed the fiber probe in colloid AuNPs (16 nm) and measured the reflection spectrum in air every 30 seconds for comparison. Figure 3b shows the reflection spectrum in air as a function of time. Figure 3b shows that AuNPs were gradually deposited onto the fiber end-face when the fiber probe was immersed in AuNPs solution. The evolution of its reflection spectra resembles that in Figure 3a. In Figure 3b, yellow arrow and purple arrow indicate the attenuation valley variation trend and the trend of the longer-wavelength strong peak shift as the number of bilayers increases, respectively. The wavelength shift of its strong peak as function of deposition time is evaluated in Figure 3c also. The difference between Figure 3a and Figure 3b lies in that the attenuation valley of Figure 3a is stronger than that of Figure 3b. Also, the wavelength shift of the strong peak in Figure 3a is larger than that in Figure 3b. Therefore, it means PAH matrix enhances the LSPR-induced attenuation which “pushes” the peak wavelength of the strong peak to longer wavelength further.

### 2.3. AFM Images of ISAM Gold Films on Fiber End-Face

**Figure 4.**
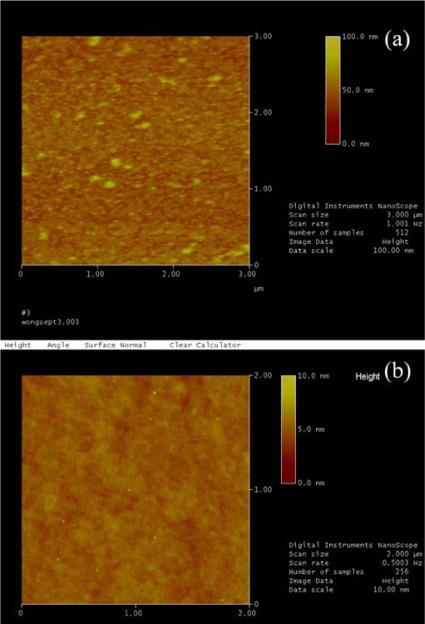
AFM images of end-face of **(a)** 12-bilayer PAH/AuNPs coated fiber probe with the 570 nm-attenuation valley in reflection spectrum, and **(b)** 20-bilayer PAH/AuNPs coated fiber probe without the 570 nm-attenuation valley in reflection spectrum.

To study the mechanism induced by the LSPR phenomenon of PAH/AuNPs (16 nm) gold films, we used Atomic force microscope (AFM) to map the morphology of the end-face of the ISAM coated fiber probe. We took the AFM images of two fiber probe samples: one sample is the 12-bilayer ISAM coated fiber probe with the strong attenuation valley at 570 nm, and the other one is the 20-bilayer ISAM coated fiber probe without the attenuation valley at 570 nm as shown in Figure 3a. Their AFM images are shown in Figure 4. We note that in Figure 4a, AFM image of the 12-bilayer ISAM fiber probe shows a high density of small particles whose height is ∼15 nm and they should be the individual AuNPs. In addition, there are some larger bumps whose height are about 30–40 nm and they may be aggregated AuNPs. In Figure 4b, AFM image of the 20-bilayer ISAM fiber probe show a rough surface but there are no clear high density of small particles. It suggests the film becomes quasi-bulk gold films. There are only some ∼5–7 nm tall tiny “dusts” and we are not sure what they are but definitely they are not 16 nm-sized AuNPs. Hence, these AFM images suggest that the attenuation valley at 570 nm results from the LSPR of the mixture of individual and aggregated AuNPs dispersing on the end-face of the fiber probe. When the film becomes quasi-bulk gold films, the LSPR peak property of individual/aggregated AuNPs will lose and the attenuation valley at 570 nm will disappear. Therefore, we think, for first several bilayers of ISAM procedure, there are more and more exposed individual/aggregated AuNPs onto the surface of end-face of fibers. It causes the 570 nm attenuation valley becomes stronger and stronger, which “pushes” the strong peak to longer wavelength. The reflection spectrum of the probe is determined by the subtraction of LSPR peak from the bare fiber reflection spectrum. Afterwards, the fiber end-face is being gradually filled with more and more AuNPs, eventually the film becomes quasi-bulk gold films. The film loses individual AuNPs’ LSPR peak property. Accordingly, the reflection spectrum is determined by the correlation between the reflectance of bulk gold film and the bare fiber reflection spectrum.

### 2.4. Simulations of ISAM Films on Fiber End-Face

**Figure 5.**
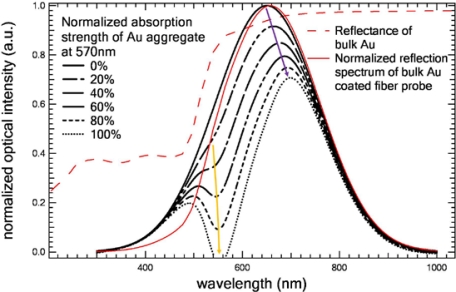
Calculated reflection spectrum of Au aggregate coated fiber probe as a function of attenuation strength of Au aggregate at 570 nm, red thin curves show reflectance of bulk Au film and normalized reflection spectrum of bulk Au film coated fiber probe. Yellow arrow and purple arrow indicate the trend of the LSPR-induced attenuation and the trend of the longer-wavelength strong peak shift as the LSPR attenuation strength increases, respectively.

To theoretically confirm our analyses, we simulated the LSPR effect of AuNPs and the reflectance effect of bulk Au film. The details of the simulation method are described in Reference 18. In our simulations, we assume, in the first several PAH/AuNPs bilayers, there is a AuNPs mixture (size from 15 nm to 45 nm) on the fiber end-face and the 570 nm attenuation valley is caused by the overall LSPR effect of this mixture. We assume that when the number of PAH/AuNPs bilayers increases, more and more AuNPs will be deposited onto the fiber end-face and the LSPR-induced attenuation at 570 nm will increase accordingly. Hence, in our simulations, the increase of the number of PAH/AuNPs bilayers (or the number of AuNPs) is represented by the increase of the LSPR-induced attenuation strength. Figure 5 shows our simulation results. As the attenuation strength increases from 0% to 100% at 570 nm, the attenuation valley becomes deeper and deeper and it splits the reflection spectrum into two peaks: one small weak peak at shorter wavelength and one big strong peak at longer wavelength. Meanwhile, it “pushes” the strong peak to longer wavelength. In addition, normalized reflection spectrum of bulk Au film coated fiber probe is calculated via multiplying the bare fiber reflection spectrum by the reflectance of bulk Au film. We noted that after shaped by the reflectance of bulk Au film, the peak wavelength of the bare fiber reflection spectrum shifts to a longer wavelength. These simulated results are consistent with our experimental results on the trend of LSPR-induced attenuation variation and the trend of the longer-wavelength strong peak shift as well as the bulk Au film (or e.g., 20-bilayer PAH/AuNPs film) reflectance spectrum. They confirm our thought that the attenuation valley at 570 nm results from the LSPR effect of the mixture of individual and aggregated AuNPs dispersing on the end-face of the fiber probe. The overall attenuation strength becomes stronger and stronger as the number of bilayers increases. After several bilayers, the fiber end-face is eventually fully filled with AuNPs and the film become quasi-bulk gold films. Finally, the reflection spectrum of the AuNPs coated fiber probe is determined by the correlation between the reflectance of bulk gold film and the bare fiber reflection spectrum.

### 2.5. Biotin-Streptavidin Immunobiosensors

**Figure 6.**
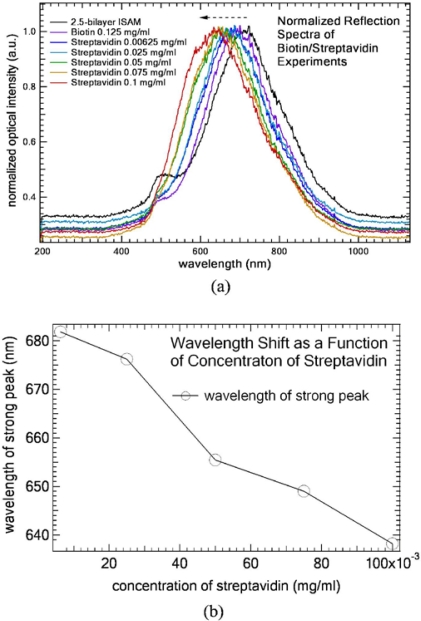
(a) Normalized reflection spectra measured in air as a function of the concentration of streptavidin solution using the same fiber probe. Black dash arrow indicates the direction of the wavelength shift of the longer-wavelength strong peak as concentration of streptavidin solution increases. (b) wavelength shift as a function of concentration of streptavidin.

Finally, we demonstrate this ISAM gold thin-films coated fiber probe for use as a biosensor by using biotin-streptavidin bioconjugate pair [16]. Biotin and streptavidin were dissolved in phosphate buffered saline (PBS) buffer at pH = 7.4 (Sigma-Aldrich Corp.) First, a 12.5-bilayers ISAM gold film (12-bilayers PAH/AuNP followed by 1 monolayer PAH) was deposited onto the fiber tip to obtain the two-peak feature. Then, the fiber probe was immersed in the biotin solution of PBS for 40 minutes to obtain biotinylated-PAH. After rinsing it thoroughly with PBS solution, the LSPR fiber probe was obtained. The probe was immersed into streptavidin solution of PBS for 15 minutes, and rinsed thoroughly by PBS solution. In our concentration experiment, the same fiber probe was used to detect streptavidin solutions of various concentrations. We monitored the wavelength shift of the longer-wavelength strong peak. Figure 6a shows the normalized reflection spectra measured in air as a function of the concentration of streptavidin solution and Figure 6b shows the corresponding wavelength shift as a function of concentration of streptavidin. Black dash arrow indicates the direction of the wavelength shift of the longer-wavelength strong peak as concentration of streptavidin solution increases. We notice (a) the small LSPR-induced attenuation at 570 nm gradually smoothes and disappears; and (b) the wavelength of the longer-wavelength strong peak red-shifts to the shorter wavelength as the concentration of streptavidin increases. In Figure 6, eventually the film surface became uniform organic films (*i.e.,* biotin-streptavidin bilayer) and the original isolated individual/aggregated AuNPs were fully buried under organic films. As such, the resulting composite film losg individual AuNPs’ LSPR peak property. The attenuation valley disappeared and the strong peak shifted back to the shorter wavelength. The sensitivity is about 465.5 nm/(mg/mL) or 0.4655 nm/(μg/mL). Given that the resolution of the spectrometer is ∼0.0001 nm and the core diameter of the fiber is 50 μm, the corresponding sensitivity is ∼800 pg/mm^2^. Compared to the sensitivity (*i.e.,* 10 pg/mm^2^) of traditional LSPR sensors based on SAM and lithography techniques [4,8], the sensitivity of this ISAM-based is only a factor of 80 poorer than the best LSPR sensitivities. In addition, its sensitivity can potentially be improved by further optimizations such as optimizing AuNPs in terms of size and shape, optimizing pH values of solutions, optimizing annealing process and so on. While retaining all of the other desirable features of LSPR sensors, the ISAM-based LSPR sensor owns several advantages such as simplicity, easy-to-fabricate, and cost-effectiveness.

## 3. Conclusions

Summarily, in this paper, we showed that the ISAM technique is a powerful technology to construct nano-architecture gold films using gold nanoparticles. Coating nm-thick ISAM gold films on optical fibers enable fine-tuning LSPR properties as fiber probes. In addition, we demonstrated that by monitoring the reflected peak shift indirectly caused by the LSPR extinction peak variation, this fiber probe can be potentially used for biosensing applications. Compared to other nanoscale thin-film techniques to build AuNPs structure such as SAM and the sputtering/lithography technique, the process of the ISAM technique is much simpler (no chemical preparation or high-energy process is needed), faster (*i.e.,* a few minutes), and cost-effective (no expensive equipment is needed). Moreover, a diverse array of materials, e.g., organic/inorganic/metal nanoparticles, macromolecules, proteins, DNA, and virus particles, can be incorporated into ISAM films. Therefore, ISAM adsorbed on optical fibers can potentially provide a simple, fast, robust, and low-cost, platform for constructing efficient fiber optic LSPR biosensors.

## References

[1] Hutter E, Fendler JH (2004). Exploitation of localized surface plasmon resonance. Advan. Mater.

[2] Kelly KL, Coronado E, Zhao LL, Schatz GC (2003). The optical properties of metal nanoparticles: The influence of size, shape, and dielectric environment. J. Phys. Chem. B.

[3] Haes AJ, Van Duyne RP (2004). A unified view of propagating and localized surface plasmon resonance biosensors. Anal. Bioanal. Chem.

[4] Mitsui K, Handa Y, Kajikawa K (2004). Optical fiber affinity biosensor based on localized surface plasmon resonance. Appl. Phys. Lett.

[5] Dhawan A, Muth JF (2006). In-line fiber optic structures for environmental sensing applications. Optics Letters.

[6] Diez A, Andres MV, Cruz JL (2001). In-line fiber-optic sensors based on the excitation of surface plasma modes in metal-coated tapered fibers. Sensor. Actuator. B-Chem.

[7] Leung A, Shankar PM, Mutharasan R (2007). A review of fiber-optic biosensors. Sensor. Actuator. B-Chem.

[8] Zhang XY, Whitney AV, Zhao J, Hicks EM, Van Duyne RP (2006). Advances in contemporary nanosphere lithographic techniques. J. Nanosci. Nanotechnol.

[9] Decher G (1997). Fuzzy nanoassemblies: Toward layered polymeric multicomposites. Science.

[10] Bertrand P, Jonas A, Laschewsky A, Legras R (2000). Ultrathin polymer coatings by complexation of polyelectrolytes at interfaces: Suitable materials, structure and properties. Macromol. Rapid. Commun.

[11] Schmitt J, Grunewald T, Decher G, Pershan PS, Kjaer K, Losche M (1993). Internal structure of layer-by-layer adsorbed polyelectrolyte films—A Neutron and X-Ray Reflectivity Study. Macromolecules.

[12] Lvov Y, Decher G, Sukhorukov G (1993). Assembly of thin-films by means of successive deposition of alternate layers of DNA and poly(allylamine). Macromolecules.

[13] Faul CFJ, Antonietti M (2003). Ionic self-assembly: Facile synthesis of supramolecular materials. Advan. Mater.

[14] Liu S, Montazami R, Liu Y, Jain V, Lin MR, Heflin JR, Zhang QM (2009). Layer-by-layer self-assembled conductor network composites in ionic polymer metal composite actuators with high strain response. Appl Phys Lett.

[15] Wang ZY, Heflin JR, Stolen RH, Ramachandran S (2005). Analysis of optical response of long period fiber gratings to nm-thick thin-film coatings. Opt. Express.

[16] Wang ZY, Heflin JR, Van Cott K, Stolen RH, Ramachandran S, Ghalmi S (2009). Biosensors employing ionic self-assembled multilayers adsorbed on long-period fiber gratings. Sensor. Actuator. B-Chem.

[17] Turkevich J, Stevenson PC, Hillier J (1951). A study of the nucleation and growth processes in the synthesis of colloidal gold. J. Discuss. Faraday Soc.

[18] Dhawan A, Muth JF (2006). Plasmon resonances of gold nanoparticles incorporated inside an optical fibre matrix. Nanotechnology.

